# Local research evidence for public health interventions against climate change in Vietnam

**DOI:** 10.3402/gha.v7.26552

**Published:** 2014-12-08

**Authors:** Joacim Rocklöv, Kim Bao Giang, Hoang Van Minh, Kristie Ebi, Maria Nilsson, Klas-Göran Sahlen, Lars Weinehall

**Affiliations:** 1 Epidemiology and Global Health, Department of Public Health and Clinical Medicine, Umeå University, Umeå, Sweden; 2 Institute for Preventive Medicine and Public Health, Hanoi Medical University, Hanoi, Vietnam; 3 School of Public Health, University of Washington, Seattle, WA, USA

The determinants of health and well-being include a wide range of environmental and social factors. Increasingly recognized drivers of injuries and ill-health are the consequences of changing weather patterns, climate extremes, and climate change. The evidence of such effects is, however, under-researched in low- and middle-income countries. For the majority of studies, originating from high-income settings, the context is considerably different. Understanding the risks better and how to manage them, from a local to a global scale, is key to future sustainable development and effective health protection policies. The domains of health risks from climate variability and change have been described in the latest assessment report from the Intergovernmental Panel on Climate Change and summarized in a recent paper (1); these include morbidity and mortality from extreme weather and climate events, infectious diseases, under-nutrition associated with changing weather patterns, and respiratory diseases associated with exposure to aeroallergens, ozone, or particulate matter.

Evidence is needed of the associations between weather and health to understand the potential negative impacts of climate variability and change; to inform adaptation strategies to prepare for, cope with, and recover from climate-change-related impacts; and to underpin local and global policies to reduce greenhouse gas emissions. Evidence and projections of the health risks of climate change are needed at all temporal scales, starting with observational evidence of the health consequences of recent changes in weather patterns. At the seasonal scale, increased understanding of climate variability, and its influence on societies and public health, is offering opportunities to develop early warning systems to protect human health. Over the longer term, model projections are needed of how climate and development patterns could interact to influence the geographic range, alter historic seasonal patterns of disease, and affect the intensity of climate-sensitive health burdens. Thus, modeling can provide essential insights into how the greenhouse gas emissions of today may affect future public health and welfare. Local health impact evidence can be a powerful tool in creating local public opinion and mandate to climate change mitigation.

Evidence across all geographical and temporal scales are largely missing for low- and middle-income countries, limiting the understanding of the magnitude and pattern of the associations between weather and climate with health outcomes. This lack of evidence means that health protection programs are typically top–down lead initiatives, such as regional disaster management plans and national climate change adaptation policy recommendations (where they exist). Although such initiatives are extremely important, health protection and climate change adaptation is an iterative process between stakeholder groups at local to international levels to ensure that policies and programs take the local context into account, thus facilitating greater efficacy and uptake. However, researchers are largely absent from these discussions, and the competence, capacity, and economic incentives for research training have been limited so far (2).

Vietnam is a country whose economy is growing strongly, with distinct demographic and epidemiological transitions and urbanization. It is characterized by large heterogeneity in the populations, with significant urban and rural differences. The climate of Vietnam is today sub-tropical to tropical with noticeable differences in landscape and considerable weather differences from north to south and from coastal to mountain regions. Projections of climate change indicate substantial changes in temperature and rainfall patterns over coming decades, as well as expected increases in extreme weather and climatic events such as floods and storms (3, 4). Vietnam has been significantly affected by extreme weather and climate events. Over the past 20 years, natural disasters have resulted in the loss of over 13,000 lives, an average annual damage loss of 1% of the gross domestic product. To increase resilience to climate variability and change, the Vietnamese government approved policies such as the 2007 National Strategy for Natural Disaster Prevention, Response and Mitigation to 2020; and the 2008 National Target Program in Response to Climate Change. The government is developing laws on climate change and disaster management policies.

This special issue is an important milestone in understanding some of the health risks of climate change by the generation of new evidence of the associations between health and weather and climate variability in Vietnam, as well as studying strategies and effectiveness in managing health protection to climate extremes. The evidence provided forms a baseline against which further changes in climate-sensitive health burdens can be measured. This evidence can also be used to augment current policies, and to develop new policies to more efficiently protect current and future populations in a changing climate.

The special issue is the product of local training of researchers in generating evidence on the health impacts of weather and climate. It is an important step in building local capacity and research competence in Vietnam on climate change and health. This work bridged research synergies and interests between partner universities in Vietnam and Umeå University, Sweden. Many research questions and needs were addressed in this special issue, with more research needed to further explore evidence and policy to serve the interest of public health.

We believe that it is not ethical, sustainable, or economically cost-effective to generate evidence of climate change impacts in low- and middle-income settings by research institutions in high-income countries without the intention to build local and national excellence in the research within the low- and middle-income institutions. The research must aim to foster mutual sustainable collaborations and development. This special issue is a product of successful north–south research collaboration.

The special issue includes eight original contributions outlining the best evidence on a range of relationships between health and weather and climate at the local level. Results show new and important evidence to understand local impacts today, and under future scenarios of climate changes. The contributions of this research include:

- The association and predictions of dengue epidemics by weather data (5, 6) using time series modeling. The studies may lead the way for setting up weather-based early warning systems for predicting infectious disease outbreak.
- The primary health care capacity to respond to storm- and flood-related ill-health in rural Vietnam using a mixed methods approach (7). We found that the primary care system capacity in rural Vietnam is inadequate for preparing for and responding to storm- and flood-related health problems in terms of preventive and treatment healthcare. National and local policies need to be strengthened and developed in a way that transfers into action in local rural communities.
- The relationship between weather and the cardiovascular hospitalization risks in northern Vietnam using sophisticated analytical non-linear time series methods (8). The study identifies susceptibility of the populations to cold exposure that must be taken seriously.
- The relationship between influenza-like illness and weather factors (9). This study highlights the complexity of the flu outbreaks and their global and local interactions with the climate regimen.
- The perceptions of climate change and health associations in subpopulations in Hanoi using a mixed methods approach (10).
- The seasonal mortality rates in Hanoi (11). This study researches the seasonal mortality peaks in Hanoi and relates the findings to previous studies from high-income settings with vastly different populations and climate regimens.
- The nutritional situation of children between 2 and 5 years of age in a northern agriculturally dominated province of Vietnam (12). The study highlights interesting differences between seasons in terms of food intake and food availability. The study may serve as a baseline for future studies of the nutritional situation of the population.

In conclusion, the studies identify important weather and climate variables associated with adverse health outcomes in Vietnam. Some results show worsened health situations when temperatures are extremely high – an event that is likely to increase in frequency with climate change. Other results show an inadequate capacity of the health care sector to respond to extreme weather and climate events; these events are expected to increase in frequency and intensity with climate change. The results of some studies facilitated the development of early warning systems by showing the relationships and accuracy of predictions of epidemic outbreaks from different weather patterns. Increased development and use of early warning systems will help protect human health now as the local historic patterns shift. However, not all of these studies show evidence of potential increasing impacts with climate change. For example, the results highlight a susceptibility of the population in northern Vietnam to cold exposure; such impacts are also of great importance for public health, and development of interventions in housing, heating, and public behavior is needed to reduce negative impacts of cold exposure. Effectiveness of interventions to protect against cold related mortality are manifested by the relatively low mortality in the winter season observed in high latitude countries such as Sweden (13, 14).

This is a first step to generate evidence, competence, and capacity needed to understand and build local resilience to the health consequences of climate change in Vietnam and beyond. More is needed for promoting resilience to the health impacts of climate change and for promoting mitigation to reduce future public health risks.
